# Can Treaties Abolish Gas Warfare?

**Published:** 1918-12-07

**Authors:** 


### The Hospital

The Workers' Newspaper of Administrative Medicine and Institutional Life, Administration, National Insurance and Health.

No. 1696, Vol. LXV. SATURDAY, DECEMBER 7, 1918. PRICE TWOPENCE

### CAN TREATIES ABOLISH GAS WARFARE?

While it must be freely admitted that according to the proclamations of history man is devoted to the art of warfare, it can equally be claimed—accepting all protests at face value—that his fondest dream is of universal peace. Great and terrible wars, not unnaturally, have again and again been followed by the production of schemes intended to render impossible the repetition of such calamities, and to this extent hope has triumphed over experience. The record is not an encouraging one in relation to our present position, and it affords considerable justification for those who set against modern proposals the leagues and alliances and treaties which have enclosed the aspirations of successive generations and have in turn yielded to the pressure of ambition or of passion. The hot fit is succeeded by the cold one, and the sinner under the sense of suffering protests that henceforth sweet reasonableness and sanctity shall be his guides. Yet the generation passes and the cycle repeats itself. The stories of heroism, of valour, and achievement are preserved and glorified, and the tales of horror, misery, and loss are forgotten. Historians, novelists, poets, painters, and orators all help in this process of inspiration and of oblivion, and youth grows up not unready for the next great adventure. Will history repeat itself? Time alone will provide a sure answer, but in view of the repeated experiences of man's record even the faith of the most enthusiastic can hardly be entirely unmixed with fears.

A lesser ambition than that which aims at the total abolition of war strives to deprive warfare of some of the worst of its evils, and if ever room existed for such an aim it is surely here and now. For four years and more tales of terror, of suffering, and of death have run round a shuddering world. We have, indeed, supped full of horrors. Not war alone has been our lot, but war rendered diabolical by the suppression of generosity and chivalry and forbearance, and by the addition of many treacherous and malignant cruelties made possible by the ingenuities of science. The low and unworthy morality of the cheap phrase which declares that all is fair in love and war has been carried into full practice, and, for the time being at all events, humanity is staggered at the demonstration. A recent letter to the *Times*, signed by the Presidents of the medical and surgical corporations of Great Britain and by the Regius Professors of Physic at the Universities of Oxford and Cambridge, is one of the evidences of this state of mind. The object of the letter is to enter a protest against the use of poisonous gases in warfare, and to claim that such a practice should be condemned by the law of nations. No doubt these sentiments will command widespread sympathy among our own people, who, with all their faults, have an instinctive and passionate attachment to what they call fair fighting. To the British mind it is not fair fighting to set free a volume of poisonous gas which will take its toll of non-combatants and combatants alike, which tends to cause its victims prolonged and terrible sufferings, and which, by a natural process of extension, may in the future be used to murder a whole town or even an entire nation. From such an action every generous and decent mind instinctively shrinks. It is, to suggest no higher motive, outside the rules of the game. The gentlemen who sign the letter to the *Times* feel all this equally with their fellow-countrymen, and hence they are sure of a general approval both of their protest and of the motives which have prompted it. Thus the letter may take its due place in the history of the war, and it has special justification seeing that some of its writers have personal experiences of the ills and agonies which the poison gases of the enemy have inflicted on British soldiers.

Unfortunately there are events even more significant and more commanding than letters to the *Times*, and one of these appears in the record that poison gases were introduced in the war by German soldiers, and that the practice was approved or even applauded by the German nation. Surely this compels the inference that public opinion in Germany is more concerned with what it judges to be effective fighting than with, as we call it, fair fighting. The Hun would agree with all the propositions stated in the *Times* letter, but would approve the poison gas all the same, and there is not the least reason to believe that this letter will convert the

German nation to the abolition of such a practice. This is the hard fact which has to be faced. The learned Presidents propose to meet it by decreeing among the peace conditions the abolition of all forms of gas warfare. Naturally they have a certain professional confidence in the value of a prescription as a cure for the ills of humanity, but in this instance the faith of the sufferers will hardly cling with confidence to the remedy. Given a repetition of war in Europe, is anyone so simple-minded as to believe that the Germans would deny themselves the use of gas as an offensive weapon because the practice had been forbidden by an international instrument to which they had pledged their word and bond? Still more, if the value and efficacy of treaties are at the level where the Presidents and professors would appear to place them, why confine their application to a relatively minor evil? Why not decree not merely the abolition of gas but still more the abolition of war? And if it be said that a ban on the latter would be useless, why propose that the former might yield to a verbal condemnation? We have every sympathy with the aims of the *Times* letter, but we doubt its practical value. Indeed, it seems to us to offer a misleading light, inasmuch as it suggests that a repetition of the horrors of the recent war can be prevented by treaties or conventions. Now what the world desires is a guarantee against all war, and one of the influences helping to urge this desire into practice is the well-founded conviction that if war again occurs the horrors of the last few years will be repeated and intensified. Whatever disturbs this conviction weakens in some measure the effort towards permanent international peace, and in so far as the Presidents' letter moves in this direction its appearance is to be deplored.

